# Regional Specific Differentiation of Integumentary Organs: Regulation of Gene Clusters within the Avian Epidermal Differentiation Complex and Impacts of SATB2 Overexpression

**DOI:** 10.3390/genes12081291

**Published:** 2021-08-23

**Authors:** Gee-Way Lin, Yung-Chih Lai, Ya-Chen Liang, Randall B. Widelitz, Ping Wu, Cheng-Ming Chuong

**Affiliations:** 1 Department of Pathology, Keck School of Medicine, University of Southern California, Los Angeles, CA 90033, USA; gwl@tmu.edu.tw (G.-W.L.); yungchihlai@gmail.com (Y.-C.L.); ycliangtaiwan@gmail.com (Y.-C.L.); widelitz@med.usc.edu (R.B.W.); ping.wu@med.usc.edu (P.W.); 2 Department of Biochemistry and Molecular Cell Biology, School of Medicine, College of Medicine, Taipei Medical University, Taipei 110301, Taiwan; 3 Integrative Stem Cell Center, China Medical University Hospital, Taichung 40447, Taiwan; 4 Institute of New Drug Development, China Medical University, Taichung 40402, Taiwan

**Keywords:** skin appendage, feather, scale, beak, evolution, Evo-Devo, EDC, β-keratins

## Abstract

The epidermal differentiation complex (EDC) encodes a group of unique proteins expressed in late epidermal differentiation. The EDC gave integuments new physicochemical properties and is critical in evolution. Recently, we showed β-keratins, members of the EDC, undergo gene cluster switching with overexpression of SATB2 (Special AT-rich binding protein-2), considered a chromatin regulator. We wondered whether this unique regulatory mechanism is specific to β-keratins or may be derived from and common to EDC members. Here we explore (1) the systematic expression patterns of non-β-keratin EDC genes and their preferential expression in different skin appendages during development, (2) whether the expression of non-β-keratin EDC sub-clusters are also regulated in clusters by SATB2. We analyzed bulk RNA-seq and ChIP-seq data and also evaluated the disrupted expression patterns caused by overexpressing SATB2. The results show that the expression of whole EDDA and EDQM sub-clusters are possibly mediated by enhancers in E14-feathers. Overexpressing SATB2 down-regulates the enriched EDCRP sub-cluster in feathers and the EDCH sub-cluster in beaks. These results reveal the potential of complex epigenetic regulation activities within the avian EDC, implying transcriptional regulation of EDC members acting at the gene and/or gene cluster level in a temporal and skin regional-specific fashion, which may contribute to the evolution of diverse avian integuments.

## 1. Introduction

Skin, composed of several region-specific differentiated epidermis forms, acts as a barrier protecting the body from external environmental assaults including biological, physical, and chemical attacks. The cornified outer layer of the skin in the stratum corneum is composed of dead keratinocytes. This epithelial layer develops at the end of epidermal differentiation due to keratinocyte keratinization and cell death [1]. A gene cluster located within the gene locus known as the epidermal differentiation complex (EDC) encodes many proteins involved in late embryonic epidermal differentiation [2]. The human EDC contains three sub-clustered families: (1) precursor proteins of the cell envelope (CE), such as involucrin (IVL), loricrin (LOR), small proline-rich proteins (SPRRs), and the late cornified envelope proteins (LCEs); (2) calcium-binding proteins (S100); (3) S100 fused type proteins (SFTPs), such as filaggrin (FLG), trichohyalin (THH) and cornulin (CRNN) [3]. The importance of the EDC is reflected by the involvement of EDC members in many human skin diseases. For example, FLG mutations lead to ichthyosis vulgaris, in which the granular layer is reduced, and hyperkeratosis is prevalent. Both characteristics are strongly associated with atopic dermatitis (AD) and AD-associated asthma [4,5,6,7,8].

The EDC locus was first identified in mammals, but the EDC locus originated from a single or small number of ancestral genes and diversified during evolution in amniotes [9,10,11,12,13]. Adaptation of the novel and complex cornified skin appendages such as scales and feathers are impacted by molecular innovations of members within the EDC genes that allowed animals to inhabit the land using a variety of terrestrial lifestyles [14,15,16,17,18]. The nomenclature of EDC genes provides the clue of distinct amino acid content, such as cysteine (C), histidine (H), proline (P), glutamic acid (E) rich, glutamine (Q) repeats, or some motifs (M). Distinctive amino acid compositions of EDC genes confer distinct physical properties of the skin appendages that express them. Originally, EDC genes evolved to form skin barriers. Later, duplication and diversification of EDC members contributed to the evolutionary success of amniotic integuments [9,19]. The feathers of birds, composed by polymerized α- or β-keratin bundles, display a wide range of physical properties. [18,20,21]. Through phylogenetic and gene organization analyses, sauropsid-specific β-keratins, also named corneous β-proteins (CBPs) [16], were suggested to be derived from a subclass of EDC genes [18]. During β-keratin evolution, the lately derived feather β-keratins are major feather components and play a key role in the evolution of feathers for flight [17].

More detailed roles of β-keratin have been investigated using the chicken model. Five β-keratin sub-clusters (*Claw*, *Feather*, *Feather-like*, *Scale*, and *Keratinocyte*) within the EDC on Chromosome 25 (Chr25) define macro-integument differences, while the feather β-keratin cluster located on Chr27 determines intra-appendage differences [22]. Recently, it has been proposed that the epigenetic regulatory mechanisms controlling expression of the EDC clusters are under epigenetic regulatory control by spatial genome organization in keratinocytes and through chromatin folding mediated by the chromatin regulator SATB1 [23,24,25,26]. Detailed analyses of the expression and regulation of β-keratins during chicken skin development in different regions (feathers vs. scales) were demonstrated in our recent study [27]. Two modes of epigenetic regulation were proposed for β-keratins located on Chr25 or Chr27 in the avian model. We found that enhancer-driven coexpression regulates the whole β-keratin sub-cluster on Chr25. However, temporospatial chromatin looping of the feather keratin cluster occurs on Chr27. CTCF/KLF4, and SATB2, molecules considered with a role to organize chromatins, may work together with competent factors such as AP-1 to build chromatin loops and then set up the region-specific β-keratin expression from Chr27 [27].

Apart from β-keratins, only a few expression patterns of conserved EDC genes have been examined in birds. These include LOR, CRNN, and SCFN (scaffoldin) [11,28,29,30] and feather-enriched EDC members such as EDCRP (epidermal differentiation cysteine-rich protein), EDDM (epidermal differentiation protein-containing DPCC motifs), EDWM (epidermal differentiation protein-containing WYDP motif), and EDMTFH (epidermal differentiation protein with an MTF motif rich in histidine) [19,30,31,32,33].

Originally, our laboratory set out to study Feather keratins, both α- and β-keratin, from a cytoskeletal perspective. It turns out β-keratins on chromosome 25 are part of the EDC complex. With the release of new genome versions and the comparisons with EDC genes in other amniotes, such as snakes, turtles, lizards and crocodiles [9,10,12,13,32], we have come to appreciate more, the roles of EDC members in the evolution and diversification of integuments. This is the motivation for us to examine further the avian non-β-keratin EDC genes and gene sub-clusters to appreciate the duplication and divergence of EDC genes. For the avian EDC locus on Chr25, we hypothesize that the regulation of both non-β-keratin and β-keratin sub-clusters are mediated by similar mechanisms involving chromatin organizers such as SATB2, or by conserved regulatory mechanisms such as being enhancer-driven. Thus, in this study we examine the expression and epigenetic regulation of non-β-keratin EDC members during avian skin appendage development. We re-analyzed bulk RNA-seq and ChIP-seq data from different skin appendages from previous studies using the newly released chicken genome version (UCSC galGal6) [27,34]. We explored (1) the systematic expression of non-β-keratin EDCs during skin development and identified the preferential expression of several EDC members in different skin appendages, and (2) whether the expression of non-β-keratin EDC sub-clusters are regulated under enhancer control or by SATB2 or both.

Our results show that the expression of EDDA sub-cluster is possibly due to enhancer-driven regulation in E14-feathers. Also, excessive SATB2-mediated disruption on the expression of the EDCRP sub-cluster in feathers and EDCH sub-cluster in beaks suggest potential regulatory roles of SATB2 on these sub-clusters. These complex epigenetic regulatory possibilities may contribute to the diversification and adaption of avian integumentary organs, and should be explored further in the future.

## 2. Materials and Methods

### 2.1. Animals and Tissues under Misexpression of SATB2

Embryos were incubated at 38 °C (SPAFAS, Preston, CT) and staged as described in [35]. Dorsal and lower beaks, dorsal back feathers, and leg scutate scales were dissected and washed in 1× cold PBS from chick E14 (HH40) and E16 (HH42) embryos. Tissue samples were collected from wild-type and SATB2-misexpressed chicken embryos, which were sacrificed in previous studies [27,34]. SATB2-misexpressed tissues were mediated by RCAS retrovirus infections, in which constructs carried the full length of the *SATB2* gene. Concentrated RCAS viruses were injected into the amniotic cavity and hind limb of E3 (HH stage 18) embryos. The phenotypes of embryos were collected at E14 and E16. Other details have been shown in [34].

### 2.2. RNA Extraction, cDNA Synthesis and Cloning of in Situ Hybridization Probes

ISH probes to selected chicken EDC genes were designed from regions with a divergent sequence in the 3′UTR region. PCR primers for EDMTF4 are 5′-CTGTGAGGATCAACCCCAGT-3′ and 5′-TGTGCCTGTACCATTCATTCA-3′(reverse); for EDCH4 are 5′-GTACACCGGCTGCCTACTGT-3′ and 5′-TGGCTCATCTACATGGTTGG- 3′(reverse); for EDPE are 5′-CAAAACCCACTGGGCTAGAG-3′ and 5′-AAAACAATTAGGGCGAAGCA-3′(reverse); for EDQREP are 5′-CACCCACTCTGTCCTGGATT-3′ and 5′-TTCAGGCTTTGTTTTCCACA- 3′(reverse); for EDDM are 5′-GGATCCCTCTGCTGTGTCTC-3′ and 5′-CTCCAACCACATCAGTGCAG-3′(reverse). cDNA from E14-feathers and E16-scales were used for the DNA template. PCR products were purified with a PCR Purification Kit and then inserted into a pDrive plasmid (Qiagen, Hilden, Germany). T7 polymerase was used to make the antisense mRNA probe (Roche, Basel, Switzerland).

### 2.3. Paraffin Sections and in Situ Hybridization (ISH)

After washing these tissues in 1× cold PBS, we fixed them in 4% paraformaldehyde in PBS at 4 °C overnight. For ISH on tissue sections, dissected skin appendages were dehydrated through an ethanol series, then transferred to xylene, embedded in paraffin wax, and sectioned to 5–7 µm. After ISH, faint eosin staining was used as a counterstain.

### 2.4. RNA-Seq Analysis

We collected and reanalyzed RNA-seq data from three different batches for dorsal back feathers, leg scales, and beaks on heads, from chicken embryonic day 7 (E7), E9, E10, E11, E12, E14, or E16 skin from previous studies, including wild-type and SATB2-overexpressing tissue [27,34,36]. RNA-Seq raw data were accessible at NCBI GEO database (accessions GSE178651, GSE136224 and GSE87250, accessed on 8 March 2021) and the corresponding sample names were collected in Supplementary Table S1. The chicken galGal6 assembly, including un-placed and unlocalized scaffolds, was downloaded from the UCSC Genome Browser [37]. The RefSeq Release 104 annotation was downloaded from the NCBI. EDC and keratin gene annotation was added into the RefSeq annotation manually [11,16,38]. Low quality of sequencing bases were trimmed, based on the Phred quality score (<20) from both of the 5′- and 3′-ends of reads. After trimming, reads were discarded if they are shorter than 30 bp for 50 bp single-end reads produced by the HiSeq 2000, or shorter than 50 bp for 75 bp single-end reads produced by the NextSeq 500. The alignment, quantification, normalization, and differential expression analysis was performed by STAR 2.7.3a [39], through Partek Flow 10.0.21.0304 (Partek Inc., St Louis, MO, USA), featureCounts in Rsubread 2.0.1 [40], trimmed mean of M values (TMM) [41], and edgeR 3.28.1 [42], respectively. In most cases, we used the default parameters. However, we only retained expressed genes if their count-per-million (CPM) values were above 1 in at least two samples. If not, they were considered no or low-expressed genes and were discarded. The exact test with a false discovery rate (FDR) < 0.05 in edgeR was set as a threshold to identify differentially expressed genes (DEG). Identified DEG belonging to EDC members in E14-feathers, E16-feathers, E16-scales, and E14-beaks are listed in Supplementary Tables S2–S5. Coverage tracks (i.e., bigWig format) were produced by deepTools 3.5.0 [43] for visualization in the Integrative Genomics Viewer (IGV) [44].

### 2.5. ChIP-Seq Analysis

The H3K27ac-, H3K4me1- and H3K4me3-ChIP-Seq data of chicken E14 dorsal feather and leg, scale, and skin tissues were downloaded from the NCBI GEO database (accession GSE136224, accessed on 8 March 2021) and the corresponding sample names were collected in Supplementary Table S6 [27]. The same method as the previous study (Table S7.4 in [27]) with the updated chicken genome (galGal6) was used to reanalyze the ChIP-Seq data. In brief, the analysis was conducted on a locally installed Galaxy platform. Raw read files (fastq.gz) were processed using tools in the following order: fastq_groomer, fastq_quality_filter, fastq_trimmer_by_quality (window size 3; min score ≥20), bowtie2 (very-sensitive), samtool_filter2 (-q 1), samtools_sort, samtools_rmdup, MACS2 callpeak (-nomodel -extsize “value obtained from the macs predictd tool”; qvalue 0.05; –bdg –broad –broad-cutoff 0.1; 1 duplicate tag is allowed), macs2_bdgcmp (-m FE), and Wig/BedGraph-to-bigWig. The tool parameters without notes were defaults. To properly generate signal tracks representing enrichment levels of histone modifications over the whole genome, we followed instructions from https://github.com/macs3-project/MACS/wiki/Build-Signal-Track, accessed on 8 March 2021 and the tool “macs2_bdgcmp” was used. Thus, the *y* axis of histone ChIP-seq tracks represents a linear scale fold enrichment (FE) of ChIP—enriched per input genomic DNA signal intensity.

## 3. Results

### 3.1. Divergent Transcriptional Profiles of non-β-Keratin EDC Genes in Different Regions of the Avian Integument

**Figure 1:**
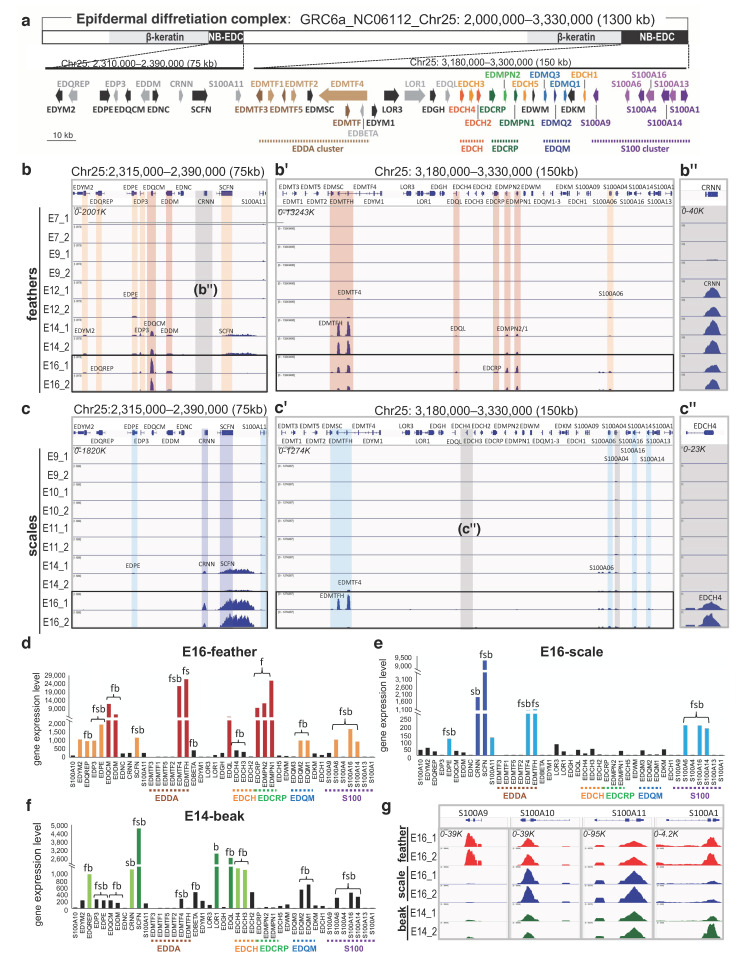
Epidermal differentiation complex (EDC) on Chr25 with different transcriptional profiles of non-β-keratin EDC (NB-EDC) members during embryonic development in feathers, scales, and beaks. (**a**) Illustration of gene organization for the non-β-EDC on Chr25. Five sub-clustered genes are highlighted here and marked with different colors; the genomic regions for β-keratin and non-β-EDC are in a fixed ratio. (**b**–**b’’**) Transcriptional profiles of feathers from E7 to E16 viewed by IGV; (**c**–**c’’**) Transcriptional profiles of scales from E9 to E16 viewed by IGV; (**d**–**f**) Gene expression level (CPM, count-per-million) of non-β-EDC in E16-feathers, E16-scales, and E14-beaks, respectively. Five sub-clusters were underlined with black bars in each panel. The expression profiles across different skin appendages (f: feather, s: scale, b: beak) were marked; (**g**) Expression levels of selected S100 genes among all skin appendages viewed by IGV. The red color marks the high expression level of EDC peaks/genes over 2000 CPM, while the orange color marks the intermediate expression level of EDC peaks/genes ranging from 500 to 2000 CPM in feathers (panel **b**,**b’**,**d**). The indigo color marks the high expression level of EDC peaks/genes over 1000 CPM, while the orange color marks the intermediate expression level of EDC peaks/genes ranging from 100 to 500 CPM in scales (panel **c**,**c’**,**e**). This analysis is based on the galGal6 genome version, while related work in our previous study is based on galGal4 [27]. Please see discussion.

In the UCSC chicken galGal6 (GRC6a) chicken genome, the EDC gene organization on Chr25 is separated into two parts within 1300 kb (Figure 1a). The 3′ region of the non-β-keratin EDC genes is mainly composed of five sub-clusters, including EDDA, EDCH, EDCRP, EDQM, and S100 (Figure 1a). Genes defined in a sub-cluster are paralogs and emerged by duplication during evolution [10]. The calcium-binding protein, S100, is a large, conserved sub-cluster among amniotes, but has only been studied in humans and mice, not in other species [3,45]. In the chicken genome, nine S100 genes are identified and seven of them form a sub-cluster located at the 3′-end of the EDC (Figure 1a). Thus, we collected expression profiles of all non-β-keratin EDC genes including the S100 family in this study (Figure 1).

During chicken embryonic development, the dorsal tracts of feathers develop 2 days prior to leg scales. For instance, feather placodes emerge from E7 in the dorsal skin, but overlapping scale placodes emerge from E9. Therefore, the RNA-seq data for feathers we reanalyzed was from E7, E9, E12, E14, and E16 (Figure 1b–b’’), whereas, the RNA-seq data for scales was from E9 to E11, E14, and E16 (Figure 1c–c’’). From these transcriptomes, we found non-β-keratin EDC genes were enriched after E14 when both the dorsal back and leg skin undergo epidermal differentiation/keratinization for distinct types of skin appendage (Figure 1b–b’’,c–c’’). Different profiles of enriched non-β-keratin EDC in feathers versus scales were also observed (Figure 1b–b’’,c–c’’,d,e). Around half of the non-β-keratin EDC genes were highly expressed in feathers at E16 (Figure 1d), but only the EDDA sub-cluster and other conserved EDC genes such as CRNN, SCFN were enriched in scales at E16 (Figure 1e).

The transcriptome profile of the E14-beak was also analyzed (Figure 1f). The distinct composition of non-β-keratin EDC in beaks compared to feathers and scales may contribute to its specialized stratum corneum and morphology (Figure 1d–f). EDCH and EDMQ sub-clusters, EDQL, LOR1, and EDQREP were preferentially expressed in E14-beaks (Figure 1f). In all non-β-keratin EDCs, only the EDCRP sub-cluster was specifically enriched in feathers, others were expressed in both feathers and scales, such as EDMTFH or in both feathers and beaks, such as EDQREP, EDQL, and EDQM (Figure 1d–f). In the S100 sub-cluster, S100A6, S100A16, S100A14 were expressed in all of the skin appendages (Figure 1d–f), but S100A9 was preferentially expressed in feathers at E16 (Figure 1g).

### 3.2. Epigenetic Analysis in Non-β-Keratin EDC Genome Regions

#### 3.2.1. Epigenetic Modification of Non-β-Keratin EDC in E14-Feathers vs. E14-Scales

**Figure 2:**
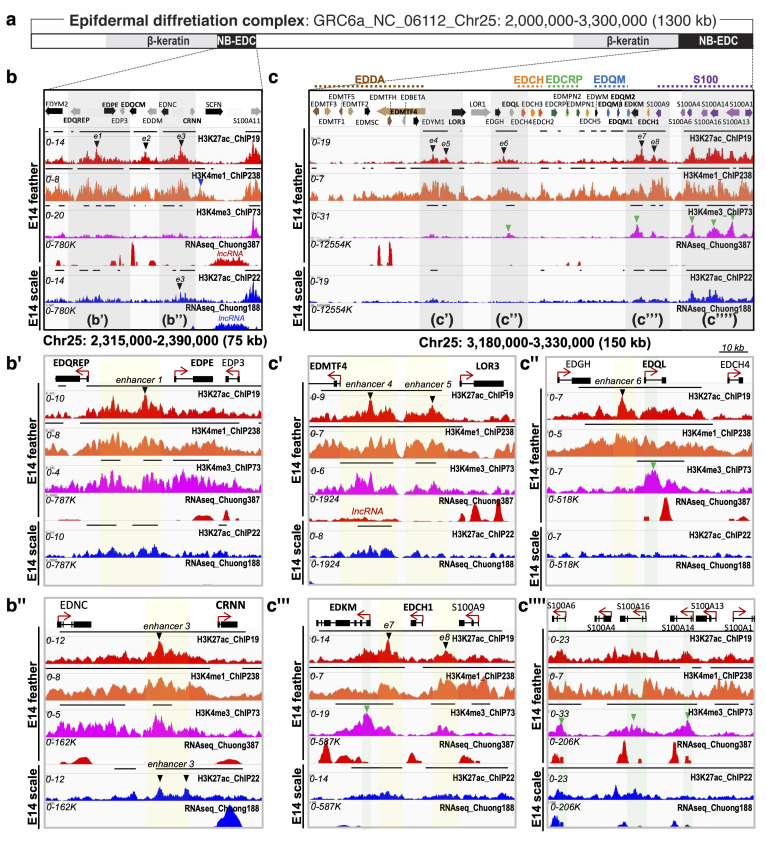
Epidermal differentiation complex on Chr25 with divergent epigenetic landscapes in E14 feathers and scales. (**a**) The locations of two parts of the non-β-EDC (NB-EDC) on Chr25 in the galGal6 genome version; (**b**–**b’’**) Profiles of RNA-seq and three chromatin marks in the N-terminal part of the non-β-EDC within 75 kb of the gene start sites. (**b’**,**b’’**) Enlargement of the two predicted enhancer regions (enhancers 1 and 3, e1 and e3, black arrowheads) from (**b**); blue arrowhead indicates the putative poised enhancer; (**c**–**c’’’’**) Profiles of RNA-seq and three chromatin marks in the C-terminal part of the non-β-EDC within 150 kb of the transcription start site. (**c’**–**c’’’’**) Enlargement of the five predicted enhancer regions (e4 to e8, black arrowheads) and the five predicted promotor regions (green arrowheads) from (**c**). The values on the y axis for ChIP-Seq data are input normalized intensities. The yellow color marks putative enhancer regions, while the green color marks the putative promoter regions. The gene name in bold may be under enhancer-mediated expression.

Chromatin modifications are useful to identify activating regulatory elements such as promoters or enhancers. Enhancers work on amplifying transcription initiation and may function on individual genes or whole gene sub-clusters. Thus, we aligned the RNA-seq data with ChIP-seq data performed with antibodies against acetylated histone H3 lysine 27 (H3K27ac), mono-methylated H3 lysine 4 (H3K4me1), and tri-methylated H3 lysine 4 (H3K4me3) in the non-β-keratin EDC genome region of E14 feathers and scales (Figure 2). Overall, many putative enhancers were predicted in E14-feathers rather than E14-scales (Figure 2b,c). For example, several putative enhancers were predicted to be active, but one located near the 5′ end of SCFN contained an H3K4me1 signal, was predicted to be inactive/poised in E14-feathers (Figure 2b, black and blue arrows, respectively). However, only one predicted active enhancer detected near the 5′ end of CRNN was identified in E14-scales (Figure 2b,b’’, black arrows), which may enhance the expression of CRNN and even SCFN (Figure 1c,e).

In E14-feathers, three presumptive active enhancers were located in the 5′-region and with the potential to regulate EDQREP or EDPE, EDQCM, and CRNN (Figure 2b–b’’). Five other presumptive active enhancers were localized in the 3′ region and with the potential to regulate EDQL and two sub-clusters (EDDA and EDQM) (Figure 2c–c’’’’). Interestingly, a lncRNA spanning around 20 kb near the 5′-end of EDMTF4 overlapped with one presumptive active enhancer (Figure 2c’). In conclusion, several putative active enhancers were identified by H3K27ac-, H3K4me1- and H3K4me3-ChIP-seq in E14-feathers rather than E14-scales, which corresponded to their enriched transcripts. Some putative active enhancers may regulate the expression of single non-β-keratin EDCs, while others have the potential to control the expression of several genes at the same time, such as the EDDA sub-cluster.

#### 3.2.2. SATB2 Is Involved in Switching the Expression of Some Sub-Clusters of the Non-β-Keratin EDC Members

**Figure 3:**
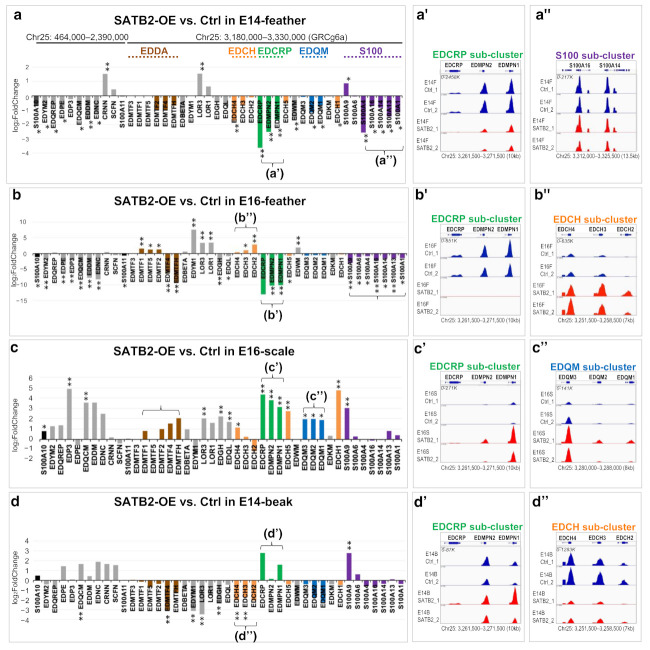
The related expression level of non-β-EDC on Chr25 when SATB2 proteins are overexpressed in different skin appendages. (**a**–**a’’**) Feathers at E14; (**b**–**b’’**) Feathers at E16; (**c**–**c’’**) Scales at E16; (**d**–**d’’**) Beaks at E14. The fold changes and statistical significance of DEG indicated in (**a**–**d**) are collected from Supplementary Tables S2–S5. DEG with an FDR < 0.05 are indicated by asterisks (*) and DEG with a Log2 fold change over 1.5 or less than −1.5, under the excess of SATB2, is marked by double asterisks (**). Five sub-clusters of EDC members (EDDA, EDCH, EDCRP, EDQM, and S100) are indicated by different colors, the same as those in Figure 1a.

Misexpression of SATB2 significantly altered α- and β-keratin expression within sub-clusters [34]. We suppose that SATB2 misexpression also works on the non-β-keratin EDC. Overexpression of SATB2 caused most non-β-keratin EDC genes to be down-regulated in E14- and E16-feathers (Figure 3a,b), while up-regulating most non-β-keratin EDC genes in E16-scales (Figure 3c). Non-β-keratin EDC sub-clustered genes were usually down/up-regulated as a unit in response to the overexpressed SATB2 proteins (Figure 3a–d, brackets). For example, the EDCRP was the most sensitive sub-cluster, which was down-regulated in feathers (Figure 3a’,b’) but up-regulated in scales when SATB2 was overexpressed (Figure 3c’). This opposite gene regulation effect was also seen in the EDCH sub-cluster in E16-feathers (up-regulation) and E14-beaks (down-regulation) (Figure 3b’’,d’’). Overall, the excess SATB2 induced down-regulation of enriched non-β-keratin EDC genes, such as the EDCRP sub-cluster in feathers and EDCH sub-cluster in beaks, in tissues with enriched endogenous SATB2. In contrast, excess SATB2 levels up-regulated most non-β-keratin EDC genes in scales, which express low levels of endogenous SATB2.

### 3.3. Expression Patterns of Several Non-β-Keratin EDC Genes and Their Disrupted Patterns When SATB2 Is Overexpressed during Epidermal Differentiation of Skin Appendages

**Figure 4:**
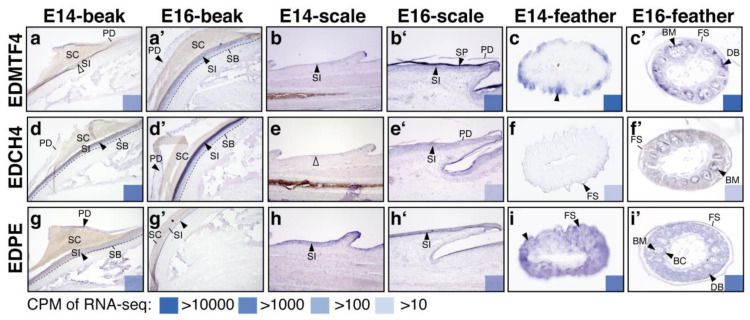
Expression patterns of selected non-β-keratin EDCs in developing beaks, scales, and feathers were analyzed by section in situ hybridization. Beaks and scales, sagittal sections. Feathers, cross sections. (**a**–**c**, **a’**–**c’**) In situ hybridization of EDMTF4 probes at E14 and E16, respectively; (**d**–**f**, **d’**–**f’**) In situ hybridization of EDCH4 probes at E14 and E16, respectively; (**g**–**i**, **g’**–**i’**) In situ hybridization of EDPE probes at E14 and E16, respectively. Filled arrowheads indicate the location of in situ hybridization signals. The expression levels (CPM, counts per million mapped reads) of each gene from RNA-seq data are indicated in the bottom right. Abbreviations: BC, barb cortex; BM, barb medulla; DB, downy barbules; FS, feather sheath; PD, periderm; SB, stratum basal; SC, stratum corneum; SI, stratum intermedium; SP, subperiderm.

According to RNA-seq results, only genes in the EDCRP sub-cluster were feather-specific (Figure 1d). In other words, there was no scale- nor beak-specific non-β-keratin EDCs. Thus, we selected a feather-and-scale enriched gene, EDMTF4, as a representative for the EDDA sub-cluster, a feather-and-beak enriched gene, EDCH4, as a representative for the EDCH sub-cluster, and an all-skin-appendage enriched gene, EDPE, to analyze their transcript patterns in beaks, scales, and feathers (Figure 4). In the beak region, transcripts of these three non-β-keratin EDCs were detected in both the periderm (PD) and stratum intermedium (SI) layers (Figure 4a,a’,d,d’,g,g’). Besides, transcripts of EDMTF4 and EDCH4 were more highly expressed in the outer layer of the stratum intermedium, which is also called the transition zone of the intermediate layer in the beak (Figure 4d,d’). In the scale region, transcripts of these three non-β-keratin EDCs were weakly expressed in the inner surface (stratum intermedium) (Figure 4b,b’,e’,h,h’), while EDMTF4 was also highly expressed in the outer surface (periderm) at E16 (Figure 4b’). Within the feather filaments, EDMTF4 was enriched in barbule cells at E14 and then restricted in the barb medulla (BM) at E16 (Figure 4c,c’). EDCH4 was weakly expressed in the feather sheath (FS) at E14 and then shifted to the barb medulla at E16 (Figure 4f,f’). However, EDPE mRNA was distributed in most of the cells of the feather filament at both E14 and E16 (Figure 4g,g’).

**Figure 5:**
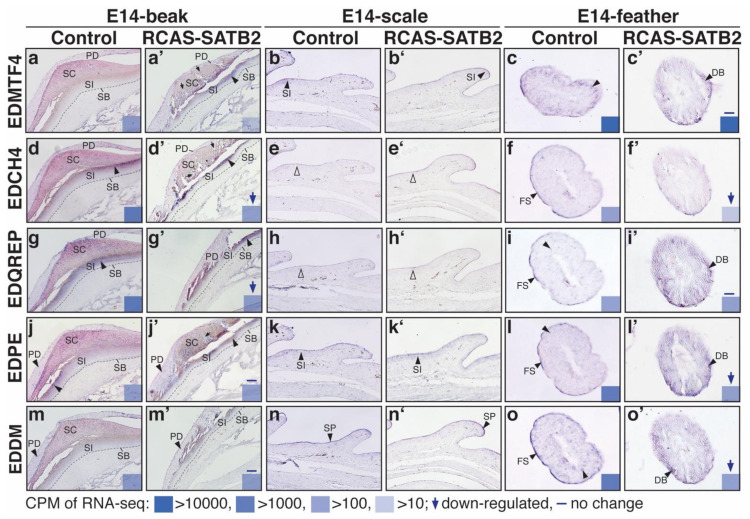
Altered expression patterns of selected non-β-keratin EDCs in beaks, scales, and feathers with SATB2-overexpression are studied by section in situ hybridization. Beaks and scales, sagittal sections. Feathers, cross sections. (**a**–**c**, **a’**–**c’**). In situ hybridization of EDMTF4; (**d**–**f**, **d’**–**f’**), EDCH4; (**g**–**i**, **g’**–**i’**), EDQREP; (**j**–**l**, **j’**–**l’**), EDPE; (**m**–**o**, **m’**–**o’**) and EDDM. Filled arrowheads indicate the location of in situ hybridization signals. The expression levels (CPM) of each gene from RNA-seq data, and disrupted trends when SATB2 is overexpressed, are indicated in the bottom right. Abbreviations: DB, downy barbules; FS, feather sheath; PD, periderm; SB, stratum basal; SC, stratum corneum; SI, stratum intermedium; SP, subperiderm.

In addition to the above three non-β-keratin EDC genes, EDQREP and EDDM were also selected to examine the disrupted pattern resulting from SATB2 overexpression (Figure 5). In our previous functional study, some missing areas are visible in the stratum corneum of the upper beak when SATB2 is overexpressed [34]. Notably, the transition zone (outer part of the stratum intermedium) becomes thinner compared to the control group [34]. We further analyzed the expression pattern of the non-β-keratin EDC in these SATB2-misexpressed tissues (Figure 5). The transcripts enriched in the stratum intermedium, such as EDMTF4, EDCH4, and EDQREP, became more condensed in the thinner transition zones (Figure 5a,a’,d,d’,g,g’,i,i’), but EDPE and EDDM that were expressed in the periderm of the upper beak at E14 kept the same expression pattern when SATB2 was overexpressed (Figure 5j,j’,m,m’). In the scale region, the abnormal phenotype produced by SATB2 overexpression was less obvious than other skin appendages [34].

Similar to the non-β-keratin EDC expression pattern, no further disrupted pattern was detected (Figure 5b,b’,d,d’, h,h’,k,k’,n,n’). Within the feather filaments at E14, transcripts of ETMTF4 were distributed in all of the barbule cells (Figure 5c), but signals for EDQREP and EDDM were only detected in partial barbule cells (Figure 5i,l,o). Besides, EDCH4, EDQREP, EDPE and EDDM were also enriched in the feather sheath at E14 (Figure 5f,i,o). The transcripts of EDQREP, EDPE, and EDDM were enriched in the advanced differentiation of downy barbules rather than the feather sheath at E14 when SATB2 was overexpressed (Figure 5i’,l’,o’). The changes in the expression levels upon overexpression of SATB2 from RNAseq were labeled in the right bottom corner of each panel in Figure 5, although the signal of in situ hybridization is not a tool for quantification. Taken together, excess SATB2 levels disrupted non-β-keratin EDC expression in the stratum intermedium of the beak and shifted their expression patterns from the feather sheath to downy barbules in some cases.

## 4. Discussion

This study demonstrates that non-β-keratin EDC members also show gene and/or gene cluster-like regulation. The clustering of EDC gene members serves as a good candidate to study whether they are able to coordinate gene regulation in a gene cluster to provide complex gene regulation at different levels (gene and gene clusters), and allow diverse expression patterns of EDC members in a temporospatial specific manner. The implication may be in parallel with the *globin* or *HOX* gene clusters [8], but there are also EDC-specific rules which we are just beginning to untangle [27].

**Figure 6:**
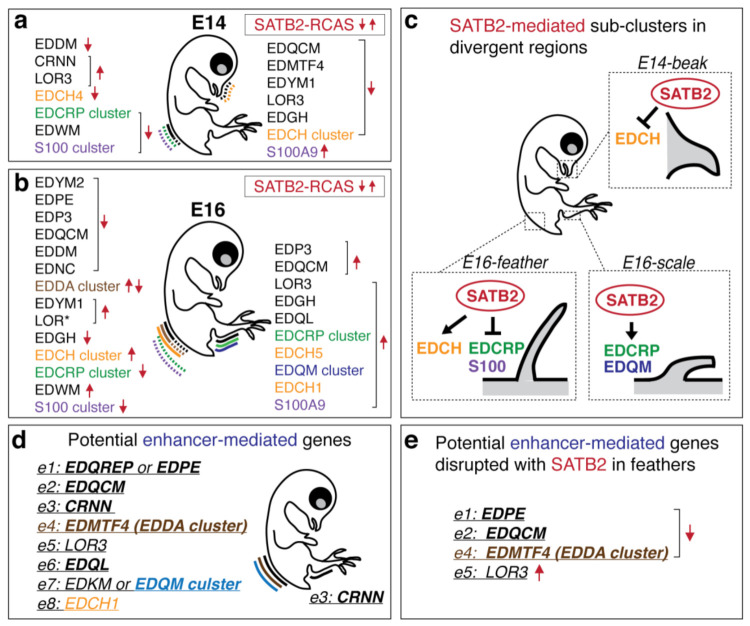
SATB2-mediated or putative enhancer-mediated expression for non-β-keratin EDC genes in different developing integumentary organs. (**a**,**b**) Summary of non-β-keratin EDC genes or gene clusters in which expression was disrupted under RCAS -SATB2 infection at E14 of feathers and beaks (**a**) or at E16 of feathers and scales (**b**) from Figure 3. Red arrows indicate candidate genes were down- or up- regulated under the SATB2-RCAS infection. (**c**) Summary of SATB2-mediated sub-clusters in divergent regions from (**a**) and (**b**). The opposite regulation of EDC sub-clusters such as EDCRP and EDCH between feathers and scales or feathers and beaks, respectively. (**d**) Summary of non-β-keratin EDC genes with putative enhancers (e1 to e8) in feather or scale regions from Figure 2. Genes in bold indicate their expression level over 500 CPMs in Figure 1. (**e**) Potential genes mediated by both SATB2 and enhancers.

In this study, we determined all transcription and epigenetic modification profiles from different stages of embryonic avian integument development focusing on the non-β-keratin genes in the epidermal differentiation complex on Chr25 (Figure 1, Figure 2, Figure 3 and Figure 6). The conserved EDC genes among amniotes, including S100 and S100 fused-type proteins, were especially enriched in scales (Figure 1e). In contrast, other later derived EDCs for avian species were enriched in feathers (EDCRP sub-cluster) or beaks (EDCH sub-cluster), respectively (Figure 1; Figure 4). We tried to identify putative active enhancers that regulate the whole sub-cluster of non-β-keratin EDCs (Figure 2; Figure 6d). All the potential genes and gene sub-clusters mediated by putative enhancers in E14-feathers detected from Figure 2 were listed in Figure 6d. Only the EDDA and EDQM sub-clusters show the possibility that they are mediated under enhancer control (Figure 6d). Interestingly, a lncRNA around 4kb transcribed from upstream of EDMTF4 also has a potential role as an enhancer RNA (eRNA) for the EDDA sub-cluster (Figure 1c’) [46,47]. However, there are technical limitations for experimental validation of predicted enhancer regions using avian species and also feather-specific enhancers which could not be verified in the mouse model. The functional assay for lncRNA through knockdown with siRNA may be available and is worth using for verification in birds because EDMTF4 is conserved across 48 bird species [19].

An excessive amount of SATB2 disrupts the expression levels of EDC genes such as the EDCRP and EDCH sub-clusters (Figure 3; Figure 6c). Similar to our previous study which demonstrated that elevated SATB2 levels targeted the EDC locus on Chr25, regulating five specific β-keratin sub-clusters (*Claw*, *Scale*, *Feather*, *Feather-like*, and *Keratinocyte*) individually [34], the skin appendages in response to excess amounts of SATB2 show regional-specific effects. The opposite gene regulation effects of non-β-keratin sub-clusters including EDCRP and EDCH under the overexpression of SATB2, between feather and scale or feather and beak, are summarized in Figure 6. We further summarized potential enhancer-mediated genes in the EDC complex, and those altered by SATB2 (Figure 6d,e). Mechanistically, how SATB2 proteins interact with potential enhancers on sub-clusters, or how SATB2 proteins work together with other cofactors to form the molecular complex and promote the transcription of gene clusters is unknown. In the mouse, the chromatin architecture of the EDC region is altered in the SATB1-null mouse epidermis [24]. In the future, we hope SATB2-ChIP-analysis can reveal the binding sites of SATB2 in the chicken EDC locus, and shine new light on the mechanism of SATB2 based chromatin organization.

Overall, the activity of gene clusters affected by SATB2 were seen in partial α-keratin sub-clusters on Chr27 and Chr33, as well as for non-β-keratin/β-keratin sub-clusters on Chr25, and the entire β-keratin cluster on Chr27 [27,34]. Although we do not know how SATB2 proteins regulate the expression of genes in the whole sub-cluster, two regulatory mechanisms have been proposed [27]. Clustered EDC gene organization is common in vertebrates from reptiles and birds to mammals [10,11]. A gene cluster regulatory mechanism may have been derived from the α-keratin sub-cluster on Chr27, when it was replicated to form the α-keratin sub-cluster on Chr33, β-keratin sub-cluster on Chr27, and EDC on Chr25 including the non-β-keratin and β-keratin sub-clusters. This clustered organization may be obtained by gene duplication of a common ancestor in evolution, but some gene replicates may have been lost. The current study provides valuable information for future studies on the epigenetic regulatory mechanism and evolution of the avian EDC.

It should be noted that in the galGal6 chicken genome, the EDC region on Chr25 is spliced into two separated parts within 1300 kb (Figure 1a). Also, the orientations of non-β-keratin EDC genes in the 3′end part are inverted in galGal6 compared to the earlier galGal4 genome version. However, gene annotation for the EDC is more complete and gene organization within two separated regions is also revised in the newly released galGal6 version (Figure 1a) [10,11,27]. To avoid confusion of each EDC gene, all the gene names in this study follow the annotation of the galGal6 version of the genome. However, if genes are unlabeled in this version of the genome, we show names associated with their LOC number in previous studies [10,11]. The combination of RNA-seq and ChIP-seq databases plus the analysis of the disrupted expression patterns when SATB2 expression levels are up-regulated suggest that SATB2 can interact with non-β-keratin EDC regions affecting the transcription of EDC gene clusters.

## 5. Conclusions

This study reveals the potential of complex epigenetic regulation activities within the avian EDC, implying transcriptional regulation of EDC members acting at the gene and/or gene cluster level in a temporal and skin regional-specific fashion, which may contribute to the evolution of diverse avian integuments.

## Data Availability

Data are contained within the article and Supplementary Tables S1–S6.

## Supplementary Materials

The following are available online at https://www.mdpi.com/article/10.3390/genes12081291/s1, Table S1: RNA-Seq sample list, Table S2: The related expression of non-β-EDC under overexpression of SATB2 in E14-feather, Table S3: The related expression of non-β-EDC under overexpression of SATB2 in E16-feather, Table S4: The related expression of non-β-EDC under overexpression of SATB2 in E16-scale, Table S5: The related expression of non-β-EDC under overexpression of SATB2 in E14-beak, Table S6: ChIP-Seq sample list.

## References

[1] Eckhart L., Lippens S., Tschachler E., Declercq W. (2013). Cell death by cornification. Biochim. Biophys. Acta (BBA)-Bioenerg..

[2] Mischke D., Korge B.P., Marenholz I., Volz A., Ziegler A. (1996). Genes Encoding Structural Proteins of Epidermal Cornification and S100 Calcium-Binding Proteins Form a Gene Complex (“Epidermal Differentiation Complex”) on Human Chromosome 1q21. J. Investig. Dermatol..

[3] Kypriotou M., Huber M., Hohl D. (2012). The human epidermal differentiation complex: Cornified envelope precursors, S100 proteins and the ‘fused genes’ family. Exp. Dermatol..

[4] Smith F.J.D., Irvine A., Terron-Kwiatkowski A., Sandilands A., Campbell L.E., Zhao Y., Liao H., Evans A.T., Goudie D.R., Lewis-Jones S. (2006). Loss-of-function mutations in the gene encoding filaggrin cause ichthyosis vulgaris. Nat. Genet..

[5] Sandilands A., Terron-Kwiatkowski A., Hull P., O’Regan G.M., Clayton T.H., Watson R.M., Carrick T., Evans A.T., Liao H., Zhao Y. (2007). Comprehensive analysis of the gene encoding filaggrin uncovers prevalent and rare mutations in ichthyosis vulgaris and atopic eczema. Nat. Genet..

[6] Henderson J., Northstone K., Lee S.P., Liao H., Zhao Y., Pembrey M., Mukhopadhyay S., Smith G.D., Palmer C., McLean W.H.I. (2008). The burden of disease associated with filaggrin mutations: A population-based, longitudinal birth cohort study. J. Allergy Clin. Immunol..

[7] Sandilands A., Sutherland C., Irvine A.D., McLean W.I. (2009). Filaggrin in the frontline: Role in skin barrier function and disease. J. Cell Sci..

[8] Segre J.A. (2006). Epidermal barrier formation and recovery in skin disorders. J. Clin. Investig..

[9] Strasser B., Mlitz V., Hermann M., Rice R.H., Eigenheer R.A., Alibardi L., Tschachler E., Eckhart L. (2014). Evolutionary Origin and Diversification of Epidermal Barrier Proteins in Amniotes. Mol. Biol. Evol..

[10] Holthaus K.B., Strasser B., Lachner J., Sukseree S., Sipos W., Weissenbacher A., Tschachler E., Alibardi L., Eckhart L. (2018). Comparative Analysis of Epidermal Differentiation Genes of Crocodilians Suggests New Models for the Evolutionary Origin of Avian Feather Proteins. Genome Biol. Evol..

[11] Davis A.C., Greenwold M.J., Sawyer R.H. (2019). Complex Gene Loss and Duplication Events Have Facilitated the Evolution of Multiple Loricrin Genes in Diverse Bird Species. Genome Biol. Evol..

[12] Holthaus K.B., Mlitz V., Strasser B., Tschachler E., Alibardi L., Eckhart L. (2017). Identification and comparative analysis of the epidermal differentiation complex in snakes. Sci. Rep..

[13] Holthaus K.B., Strasser B., Sipos W., Schmidt H., Mlitz V., Sukseree S., Weissenbacher A., Tschachler E., Alibardi L., Eckhart L. (2015). Comparative Genomics Identifies Epidermal Proteins Associated with the Evolution of the Turtle Shell. Mol. Biol. Evol..

[14] Alibardi L. (2003). Adaptation to the land: The skin of reptiles in comparison to that of amphibians and endotherm amniotes. J. Exp. Zool..

[15] Chuong C.M., Nickoloff B.J., Elias P.M., Goldsmith L.A., Macher E., Maderson P.A., Sundberg J.P., Tagami H., Plonka P.M., Thestrup-Pederson K. (2002). Contoversies in Experimental Dermatology. Exp. Dermatol..

[16] Holthaus K.B., Eckhart L., Valle L.D., Alibardi L. (2018). Review: Evolution and diversification of corneous beta-proteins, the characteristic epidermal proteins of reptiles and birds. J. Exp. Zool. Part. B Mol. Dev. Evol..

[17] Greenwold M.J., Sawyer R.H. (2010). Genomic organization and molecular phylogenies of the beta (β) keratin multigene family in the chicken (Gallus gallus) and zebra finch (Taeniopygia guttata): Implications for feather evolution. BMC Evol. Biol..

[18] Greenwold M.J., Bao W., Jarvis E.D., Hu H., Li C., Gilbert M.T.P., Zhang G., Sawyer R.H. (2014). Dynamic evolution of the alpha (α) and beta (β) keratins has accompanied integument diversification and the adaptation of birds into novel lifestyles. BMC Evol. Biol..

[19] Davis A., Greenwold M. (2021). Evolution of an Epidermal Differentiation Complex (EDC) Gene Family in Birds. Genes.

[20] Haake A.R., König G., Sawyer R.H. (1984). Avian feather development: Relationships between morphogenesis and keratinization. Dev. Biol..

[21] Alibardi L. (2017). Review: Cornification, morphogenesis and evolution of feathers. Protoplasma.

[22] Wu P., Ng C.S., Yan J., Lai Y.-C., Chen C.-K., Lai Y.-T., Wu S.-M., Chen J.-J., Luo W., Widelitz R.B. (2015). Topographical mapping of α- and β-keratins on developing chicken skin integuments: Functional interaction and evolutionary perspectives. Proc. Natl. Acad. Sci. USA.

[23] Gdula M., Poterlowicz K., Mardaryev A., Sharov A.A., Peng Y., Fessing M.Y., Botchkarev V.A. (2013). Remodeling of Three-Dimensional Organization of the Nucleus during Terminal Keratinocyte Differentiation in the Epidermis. J. Investig. Dermatol..

[24] Fessing M., Mardaryev A., Gdula M., Sharov A.A., Sharova T., Rapisarda V., Gordon K., Smorodchenko A., Poterlowicz K., Ferone G. (2011). p63 regulates Satb1 to control tissue-specific chromatin remodeling during development of the epidermis. J. Cell Biol..

[25] Botchkarev V.A., Gdula M., Mardaryev A., Sharov A.A., Fessing M.Y. (2012). Epigenetic Regulation of Gene Expression in Keratinocytes. J. Investig. Dermatol..

[26] Poterlowicz K., Yarker J.L., Malashchuk I., Lajoie B.R., Mardaryev A.N., Gdula M., Sharov A.A., Kohwi-Shigematsu T., Botchkarev V.A., Fessing M.Y. (2017). 5C analysis of the Epidermal Differentiation Complex locus reveals distinct chromatin interaction networks between gene-rich and gene-poor TADs in skin epithelial cells. PLoS Genet..

[27] Liang Y.-C., Wu P., Lin G.-W., Chen C.-K., Yeh C.-Y., Tsai S., Yan J., Jiang T.-X., Lai Y.-C., Huang D. (2020). Folding Keratin Gene Clusters during Skin Regional Specification. Dev. Cell.

[28] Alibardi L., Mlitz V., Eckhart L. (2015). Immunolocalization of Scaffoldin, a Trichohyalin-Like Protein, in the Epidermis of the Chicken Embryo. Anat. Rec. Adv. Integr. Anat. Evol. Biol..

[29] Mlitz V., Strasser B., Jaeger K., Hermann M., Ghannadan M., Buchberger M., Alibardi L., Tschachler E., Eckhart L. (2014). Trichohyalin-Like Proteins Have Evolutionarily Conserved Roles in the Morphogenesis of Skin Appendages. J. Investig. Dermatol..

[30] Alibardi L., Eckhart L. (2021). Immunolocalization of epidermal differentiation complex proteins reveals distinct molecular compositions of cells that control structure and mechanical properties of avian skin appendages. J. Morphol..

[31] Lachner J., Ehrlich F., Mlitz V., Hermann M., Alibardi L., Tschachler E., Eckhart L. (2019). Immunolocalization and phylogenetic profiling of the feather protein with the highest cysteine content. Protoplasma.

[32] Strasser B., Mlitz V., Hermann M., Tschachler E., Eckhart L. (2015). Convergent evolution of cysteine-rich proteins in feathers and hair. BMC Evol. Biol..

[33] Alibardi L., Holthaus K.B., Sukseree S., Hermann M., Tschachler E., Eckhart L. (2016). Immunolocalization of a Histidine-Rich Epidermal Differentiation Protein in the Chicken Supports the Hypothesis of an Evolutionary Developmental Link between the Embryonic Subperiderm and Feather Barbs and Barbules. PLoS ONE.

[34] Lin G., Liang Y., Wu P., Chen C., Lai Y., Jiang T., Haung Y., Chuong C. (2021). Regional specific differentiation of integumentary organs: SATB2 is involved in α- and β-keratin gene cluster switching in the chicken. Dev. Dyn..

[35] Hamburger V., Hamilton H.L. (1951). A series of normal stages in the development of the chick embryo. J. Morphol..

[36] Wu P., Yan J., Lai Y.-C., Ng C.S., Li A., Jiang X., Elsey R.M., Widelitz R., Bajpai R., Li W.-H. (2017). Multiple Regulatory Modules Are Required for Scale-to-Feather Conversion. Mol. Biol. Evol..

[37] Lee C.M., Barber G.P., Casper J., Clawson H., Diekhans M., Gonzalez J.N., Hinrichs A., Lee B.T., Nassar L.R., Powell C.C. (2019). UCSC Genome Browser enters 20th year. Nucleic Acids Res..

[38] Ng C.S., Wu P., Fan W.-L., Yan J., Chen C.-K., Lai Y.-T., Wu S.-M., Mao C.-T., Chen J.-J., Lu M.-Y.J. (2014). Genomic Organization, Transcriptomic Analysis, and Functional Characterization of Avian α- and β-Keratins in Diverse Feather Forms. Genome Biol. Evol..

[39] Dobin A., Davis C.A., Schlesinger F., Drenkow J., Zaleski C., Jha S., Batut P., Chaisson M., Gingeras T.R. (2013). STAR: Ultrafast universal RNA-seq aligner. Bioinformatics.

[40] Liao Y., Smyth G.K., Shi W. (2014). featureCounts: An efficient general purpose program for assigning sequence reads to genomic features. Bioinformatics.

[41] Robinson M.D., Oshlack A. (2010). A scaling normalization method for differential expression analysis of RNA-seq data. Genome Biol..

[42] Robinson M.D., Smyth G.K. (2007). Small-sample estimation of negative binomial dispersion, with applications to SAGE data. Biostatistics.

[43] Ramírez F., Dündar F., Diehl S., Grüning B., Manke T. (2014). deepTools: A flexible platform for exploring deep-sequencing data. Nucleic Acids Res..

[44] Thorvaldsdóttir H., Robinson J.T., Mesirov J.P. (2013). Integrative Genomics Viewer (IGV): High-performance genomics data visualization and exploration. Brief. Bioinform..

[45] Gene Expression Database (GXD), Mouse Genome Informatics Web Site World Wide Web. http://www.informatics.jax.org.

[46] Arnold P.R., Wells A.D., Li X.C. (2020). Diversity and Emerging Roles of Enhancer RNA in Regulation of Gene Expression and Cell Fate. Front. Cell Dev. Biol..

[47] Carullo N., Iii R.P., Simon R.C., Soto S.A.R., Hinds J.E., Salisbury A.J., Revanna J.S., Bunner K.D., Ianov L., Sultan F.A. (2020). Enhancer RNAs predict enhancer–gene regulatory links and are critical for enhancer function in neuronal systems. Nucleic Acids Res..

